# Defect engineered unzipped multiwalled carbon nanotube/vanadium pentoxide composite for high-performance supercapacitor application

**DOI:** 10.1039/d6ra00149a

**Published:** 2026-02-27

**Authors:** V. P. Aswathi, Vidya Raman, P. B. Sreeja

**Affiliations:** a Centre for Renewable Energy and Environmental Sustainability, Department of Chemistry, CHRIST University Bengaluru Karnataka 560029 India; b Department of Chemistry, T. M Jacob Memorial Government College Manimalakkunu Koothattukulam Kerala 686662 India Sreeja.pb@christuniversity.in

## Abstract

In the pursuit of next-generation energy storage systems, the advancement of high-performance electrode materials with enhanced capacitance and durability remains critical. This study presents a binary composite of unzipped multi-walled carbon nanotubes (UzMWCNTs) integrated with vanadium pentoxide (V_2_O_5_). The unzipping process introduces surface defects and oxygen functional groups, which enhance dispersion and provide numerous active sites. V_2_O_5_ nanoparticles uniformly anchor onto the UzMWCNT surface, offering pseudocapacitive behavior and boosting redox activity. The synergistic interaction between electric double-layer capacitance and faradaic charge storage delivers superior electrochemical performance. Structural and morphological characterization confirms successful composite formation, while electrochemical evaluations reveal a specific capacitance of 1135 F g^−1^ and cycling stability with 88% retention over 2000 cycles. This work highlights the potential of UzMWCNT/V_2_O_5_ hybrids as promising candidates for high-efficiency, next-generation supercapacitor electrodes.

## Abstract 2

Design and development of advanced high-performance electrode materials enabling high performance for next generation energy storage.

## Graphical abstract

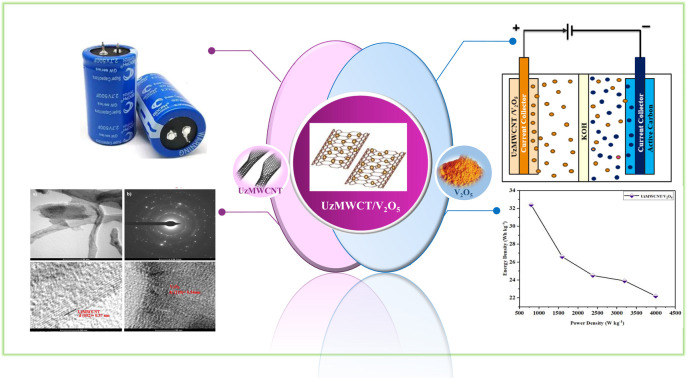

## Introduction

The increasing global focus on environmental sustainability and the critical shift towards renewable energy sources have heightened the demand for advanced energy storage solutions. Supercapacitors (SCs) have gained significant attention as potential candidates, owing to their attractive properties such as high-power densities (PD), fast charge–discharge capabilities, long operational lifespans, low fabrication cost, and greater benignity over conventional energy storage systems. Such features render SCs very appropriate for a range of applications, from mobile electronic devices to electric vehicles.^1–3^ SCs are generally classified into two categories according to their charge storage mechanisms. The first category is electric double-layer capacitors (EDLCs), in which energy is stored by the adsorption of anions and cations. The second category is pseudo-capacitors, in which energy is stored *via* rapid reactions.^4^ The electrode materials in EDLCs are mainly composed of carbon-based nanomaterials, such as carbon nanotubes (CNTs), graphene, and carbon nanofibers. Since these materials do not undergo chemical changes during charge and discharge cycles, they have superior cycling stability.^5,6^ Further, the capacitance of EDLCs is primarily determined by the specific surface area of the materials, whereas pseudocapacitive materials, like transition metal oxides (V_2_O_5_, MnO_2_, RuO_2_)^7,8^ and conductive polymers, emphasize their superior specific capacitance (*C*_s_) and energy density (ED). The interfacial area between the material and the electrolyte solution plays a crucial role in the capacitance of the materials, with larger interfaces improving energy conversion. Pseudocapacitive materials exhibit significantly higher capacitance values compared to EDLC materials.^9^ Despite this, the interface undergoes periodic compositional and structural changes with charge–discharge cycles due to the faradaic mechanisms at the interface, thus resulting in the limited cycling stability of pseudocapacitive materials. This challenge has led to the integration of EDLC and pseudocapacitive material technologies, merging their strengths for better capacitive performance.

CNTs have also attracted much interest in recent years with their exceptional and unique properties, suitable for use in batteries, fuel cells, and SCs.^10^ Their application as electrode materials in SCs is specifically considerable, attributed to their extensive surface area, superior conductive properties, and specialized pore structure.^11^ However, MWCNTs have lower Cs because of their unique morphology, characterized by concentric chambers, and the slower diffusion of ions inside internal tubes.^12,13^ Extensive research has been directed to maximize the inherent properties of CNTs; recent research has focused on surface modifications through defect engineering. Techniques such as functionalization or unzipping of the CNT surface have been utilized to alter their physical and chemical characteristics.^14^ Unzipping of MWCNTs results in the formation of the partially curved graphene structures, characterised by crumbled, wrinkled morphology and out-of-plane curvature. These properties effectively inhibit aggregation and enhance electrolyte ion penetration. Additionally, they also demonstrate enhanced dispersion stability in polar solvents and greater electrochemical reactivity compared to untreated pristine MWCNTs. The synthesis of highly dispersible UzMWCNTs involves chemically etching their surfaces. This process introduces oxygen-containing functional groups and causes unzipping or exfoliation of the MWCNT sidewalls.^15^

Among various transition metal oxides (TMOs), vanadium pentoxide (V_2_O_5_) has garnered significant interest as a highly potential cathode material for SCs, due to its high theoretical capacitance, straightforward synthesis process, low toxicity, and wide voltage window (up to 2.8 V)^16,17^ and also it exhibits multielectron redox reactions (V^5+^/V^4+^/V^3+^ couples), enabling higher ED.^18,19^

Jian Shan Ye and colleagues studied an electrochemical approach involving MWCNT in acidic media. Their findings showed a significant increase in *C*_s_, from 32.7 to 335.2 F g^−1^, when comparing pure MWCNT with oxidized MWCNT.^20^ Krishnaveni *et al.* developed an ultrasound-assisted technique to synthesize UzMWCNT/TiO_2_. The resulting structure featured UzMWCNT adorned with TiO_2_ nanoparticles, facilitating enhanced charge transfer and achieving a Cs of 475 F g^−1^.^12^ A study by Huyang *et al.* developed a ternary rGO/UzMWCNT/PANI composite featuring a distinctive three-dimensional (3D) structure, where PANI is interwoven within the rGO/UzMWCNT framework. The electrochemical testing revealed that the rGO/UzMWCNT/PANI composite demonstrated a *C*_s_ of 359.3 F g^−1^.^9^ The literature suggests that the high *C*_s_ of UzMWCNTs is attributed to their large surface area, improved functional groups, and redox activity, highlighting surface modification as key to unzipping MWCNTs into nanosheet structures for enhanced capacitance.

Recently, our group introduced a modified Hummers' method to simultaneously cut and unzip multi-walled carbon nanotubes (MWCNTs), creating unzipped MWCNTs (UzMWCNT) with enhanced structural properties combining 1D nanotube and graphene-like features. The resulting UzMWCNT/polypyrrole composite demonstrated exceptional SC performance with a *C*_s_ of 944 F g^−1^ and also exhibited excellent cycling stability, maintaining 92% of its initial capacitance following 5000 GCD cycles, highlighting the potential of controlled MWCNT modifications in developing advanced energy storage solutions.^21,22^ In another research, a ternary hybrid composite of UzMWCNT/NiFe_2_O_4_/PANI was developed. The study highlights a synergistic composite of UzMWCNT, NiFe_2_O_4_, and PANI, delivering outstanding electrochemical performance with a high Cs of 1022 F g^−1^, 84% retention after 2000 cycles in a symmetric SC.^23^

This paper reports a binary hybrid composite UzMWCNT/V_2_O_5_ that leverages the synergistic effect between electrochemical-layer capacitance and pseudo-capacitance materials. Electrochemical studies demonstrate the enhanced electrochemical performance of the composite.

## Synthesis

### Materials

Chemical reagents, including multiwalled carbon nanotube (MWCNT), vanadyl acetyl acetonate, hydrogen peroxide, hydrochloric acid, sulphuric acid, potassium permanganate, sodium borohydride, sodium nitrate, potassium hydroxide, and ethanol, were purchased from Sigma Aldrich.

### Synthesis of unzipped multiwalled carbon nanotube (UzMWCNT)

Oxidised MWCNTs were prepared *via* a modified Hummers' method. Initially, 2 g of MWCNTs were reacted with 98 mL of concentrated H_2_SO_4_, followed by the addition of sodium nitrate (NaNO_3_). The mixture was stirred for 1 hour in an ice bath. Subsequently, 6 g of KMnO_4_ was introduced as the oxidising agent and stirred for another hour under the same cooling conditions. To this oxidising mixture, 92 mL of distilled water was introduced, followed by continuous stirring for 30 minutes to facilitate further oxidation. Then, 20 mL of H_2_O_2_ and 280 mL of distilled water were added. Further impurities were removed by repeated washing using 10% HCl.

Considering that excessive oxygen functionalities on oxidised MWCNTs can adversely affect their electrical performance, a post-treatment step was employed to reduce these groups. Specifically, 500 mg of sodium borohydride was added to the O-MWCNTs and stirred for 24 hours at room temperature. The resulting product was thoroughly washed with ethanol and water to obtain purified, Unzipped MWCNTs (UzMWCNT) suitable for further application. The formation of UzMWCNT is further confirmed by the structural and morphological characterization techniques.^24,25^

### Synthesis of hybrid composite of UzMWCNT/V_2_O_5_

**Fig. 1:**
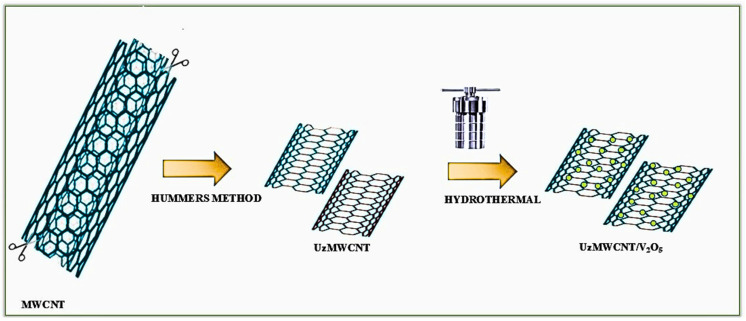
Schematic illustration of the synthesis of UzMWCNT/V_2_O_5_ hybrid composite *via* Hummer's method-based functionalization and subsequent hydrothermal treatment.

Hybrid composite of UzMWCNT/V_2_O_5_ was prepared *via* a hydrothermal method. Initially, vanadium pentoxide was synthesised from vanadyl acetylacetonate through a calcination process. The synthesised vanadium pentoxide was subsequently introduced to an ultrasonicated solution of UzMWCNTs and stirred for 1 hour. The resulting mixture was subjected to a high-temperature furnace at 300 °C for 2 hours. The final product was repeatedly washed using distilled water to remove residual impurities and obtain a purified UzMWCNT/V_2_O_5_ hybrid composite suitable for further application. Fig. 1 depicts the synthesis of the binary hybrid composite of UzMWCNT/V_2_O_5_.

### Characterization

Crystallographic information of MWCNT, UzMWCNT, and UzMWCNT/V_2_O_5_ was obtained through X-ray diffraction (XRD) analysis, performed with a Bruker AXS Kappa Apex system employing Cu Kα radiation (*λ* = 1.5418 Å) over a 2*θ* range of 10° to 80°. Raman spectroscopy was conducted using a LabRAM HR FT-Raman setup equipped with a 532 nm excitation laser. The morphological and surface characteristics of the samples were examined using a scanning electron microscope (SEM, EVO LS 15) and a transmission electron microscope (TEM, JEM-2100 Plus). Elemental analysis and chemical bonding states were investigated *via* X-ray photoelectron spectroscopy (XPS) using a ULVAC-PHI PHI5000 model probe III. A Micromeritics 3 Flex instrument was employed to determine the surface area and porosity of the materials.

Electrochemical characterization was evaluated through cyclic voltammetry (CV), galvanostatic charge–discharge (GCD), and electrochemical impedance spectroscopy (EIS), with a CHI608E potentiostat.

### Electrode preparation and symmetric supercapacitor fabrication

In a three-electrode setup, a platinum wire functions as the counter electrode, while Ag/AgCl (saturated KCl) serves as the reference electrode. The working electrode was prepared by coating nickel foam with the active material using the doctor blade technique. The electrode blend includes 85% active material, 10% polyvinylidene fluoride (PVDF), and 5% carbon black. Mixing these components with *N*-methyl pyrrolidone (NMP) yields a smooth and uniform slurry, which is applied evenly across the nickel foam. After coating, the electrodes undergo drying in a hot air oven maintained at 75 °C for 7 h.

To fabricate the symmetric SC device, identical electrodes composed of UzMWCNT/V_2_O_5_ material are assembled at the opposite terminals of the device. 1 M aqueous potassium hydroxide solution is used as the electrolyte.

## Results and discussions

**Fig. 2:**
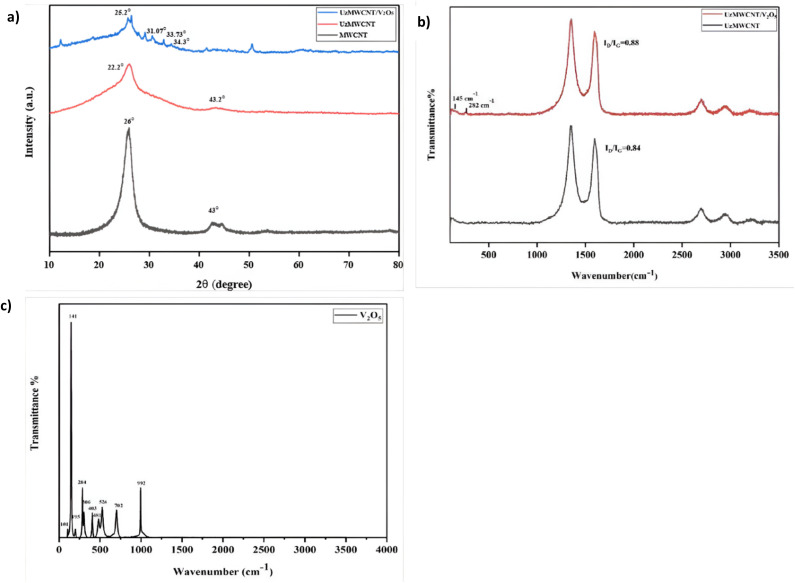
Comparative (a) XRD and (b) Raman spectra of pristine MWCNT, UzMWCNT, and UzMWCNT/V_2_O_5_, highlighting structural modifications and phase purity, and characterstic D and G bands of UzMWCNT along with Raman features of (c) V_2_O_5_.

The X-ray diffraction analysis was employed to examine the material's crystalline structure. The XRD measurements provide details of the arrangement and orientation of atoms in the crystalline phases of the material. Fig. 2a presents the XRD patterns comparing pristine MWCNT, UzMWCNT, and UzMWCNT/V_2_O_5_ hybrid composite. Pristine MWCNTs show well-defined diffraction peaks at 26° and 43°, indicative of the (002) and (100) planes, respectively.^26^ In contrast, the peaks of UzMWCNT shift, and a broader peak is observed at 23.2° and 43.2°, indicating the turbostratic disorder of UzMWCNT, confirming the unzipping of multiwalled carbon nanotubes. Upon incorporation of V_2_O_5_ in UzMWCNT, the characteristic peaks of V_2_O_5_ appear at 21.71°, 26.19°, 31.07°, and 34.3°, aligning with pristine XRD spectra of V_2_O_5_ (Fig. S.1), corresponding to (101), (110), (301), and (310) planes^27,28^ confirming the successful synthesis of the hybrid binary composite.

Raman spectroscopy was employed for characterizing the bonding arrangements of carbon atoms, specifically distinguishing between sp^3^-and sp^2^-hybridized states within the material. Its high sensitivity enables precise identification of structural variations in carbon-based materials, while functionalisation. The Raman analysis in Fig. 2b and c presents a distinct vibrational feature of V_2_O_5_ spectra, confirming the orthorhombic V_2_O_5_ phase, characterized by peaks at 101, 141, 195, 284, 306, 403, 481, 526, 702, and 992 cm^−1^. The 141 cm^−1^ mode signifies the layered orthorhombic structure, while the 195 cm^−1^ peak corresponds to V–O stretching with B_1_g symmetry. Additionally, peaks at 284, 306, and 403 cm^−1^ are attributed to V=O bond bending with B_2_g and Ag symmetry.^27,29,30^ Furthermore, Raman analysis of UzMWCNT reveals that UzMWCNT displays a more pronounced D peak compared to the G band, indicating the transformation of sp^2^ bonds into sp^3^ hybridization, clear evidence of the unzipping process occurring in the MWCNTs.^31^ In the UzMWCNT/V_2_O_5_ composite spectra, characteristic peaks of V_2_O_5_ are not evident due to the reduced intensity. The D band reflects morphological irregularities and defects in UzMWCNT, while the G band confirms the retention of sp^2^-bonded carbon within the MWCNT framework. Notably, the D-band to G-band intensity ratio (*I*_D_/*I*_G_) exhibits a slight increase from 0.84 in UzMWCNT to 0.88 in the UzMWCNT/V_2_O_5_ composite, indicating enhanced structural defects that contribute to improved capacitive performance. This integration of vibrational characteristics and structural analysis highlights the successful incorporation of V_2_O_5_ into the UzMWCNT framework.

**Fig. 3:**
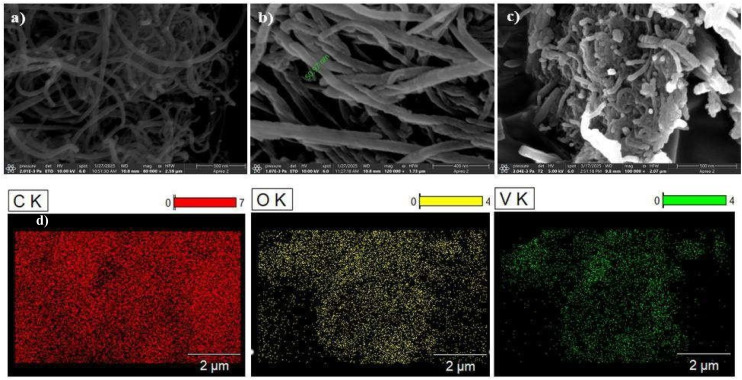
(a) FESEM micrographs of (a) MWCNT, (b) UzMWCNT, (c) UzMWCNT/V_2_O_5_, and (d) elemental analysis of UzMWCNT/V_2_O_5_ composite.

The FESEM micrographs of MWCNT, UzMWCNT, and MWCNT/V_2_O_5_ are presented in Fig. 3. Fig. 3a illustrates the well-defined tubular morphology of MWCNT. The unzipping process *via* the Hummers' method results in an increased diameter of UzMWCNT compared to pristine MWCNT, thereby enhancing the available surface area for ion interactions within the UzMWCNT framework (Fig. 3b). Fig. 3c reveals the presence of irregularly shaped V_2_O_5_ particles distributed on the surface of UzMWCNT, which effectively mitigates agglomeration and subsequently improves ion transport properties, leading to enhanced ionic conductivity. SEM-EDAX and elemental mapping analyses have been conducted to verify the incorporation of V_2_O_5_ in the synthesized composite. The elemental distribution maps for UzMWCNT/V_2_O_5_ (Fig. 3d) demonstrate the uniform presence of C, V, and O within the designated regions, further corroborating the successful integration of V_2_O_5_ into the carbon framework.

**Fig. 4:**
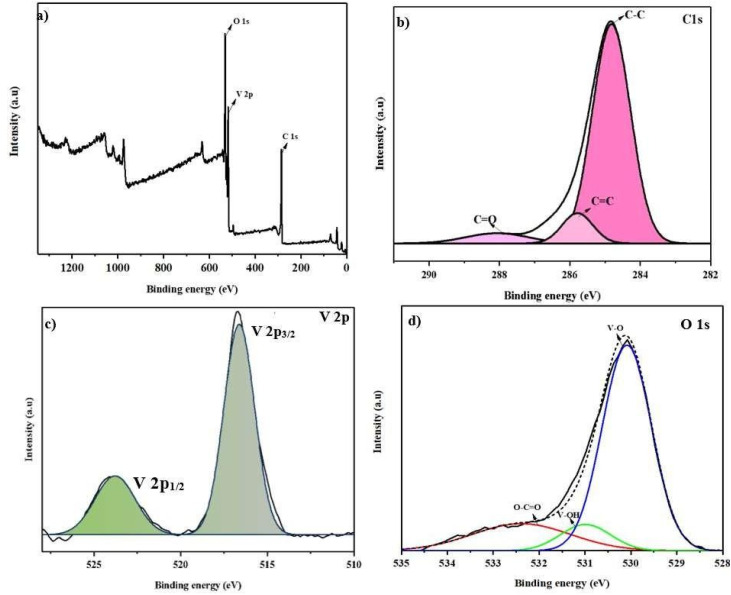
XPS analysis of UzMWCNT/V_2_O_5_ hybrid composite: (a) survey spectrum confirming successful composite formation, (b) C 1s spectrum showing graphitic carbon and functional groups from Hummer's treatment, (c) V 2p spectrum demonstrating V^5+^ oxidation state, and (d) O 1s spectrum indicating oxygen bonding configurations.

XPS analysis was employed to gain insight into the chemical environment and binding energies of the synthesized hybrid material. Fig. 4a presents the overall spectral profile of the sample, while Fig. 4b–d displays the deconvoluted elemental spectra, providing a detailed representation of the material's composition. The deconvoluted C 1s spectrum of the UzMWCNT/V_2_O_5_ composite reveals three distinct peaks. The peak at 284.8 eV corresponds to C–C bonds, while the one at 285.6 eV is associated with C=C bonding. Additionally, a peak at 287.9 eV signifies the presence of C=O bonds, providing insights into the chemical structure and bonding characteristics of the hybrid material (Fig. 4b).^32^

The high-resolution V 2p spectrum is presented in Fig. 4c displays two prominent peaks at 517.15 eV and 524.40 eV, corresponding to the V 2p_3/2_ and V 2p_1/2_ states of V^5+^, respectively.^11^ These spectral features confirm the oxidation state and electronic structure of vanadium in the composite material. Fig. 4d presents the O 1s peaks, exhibiting three distinct peaks at binding energies of 532.2 eV, 531.2 eV, and 530.2 eV, which are attributed to the presence of O–C=O groups, vanadium-bound hydroxyls (V–OH), and vanadium-oxygen bonds (V–O), respectively.^33^

**Fig. 5:**
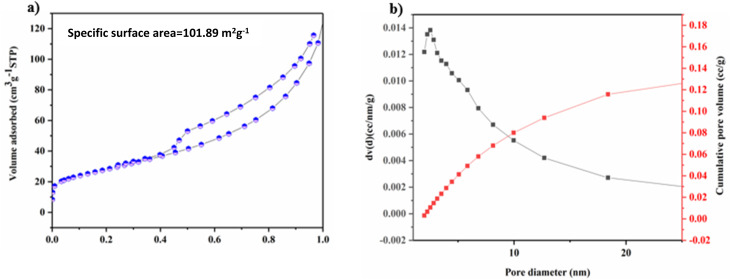
(a) Nitrogen adsorption–desorption isotherm of UzMWCNT/V_2_O_5_ (b) BJH Plot of UzMWCNT/V_2_O_5_.

The textural properties of UzMWCNT/V_2_O_5_ were examined using nitrogen adsorption–desorption isotherm analysis, as shown in Fig. 5a. The isotherm exhibits a type IV hysteresis loop within the relative pressure range of 0.48–0.99, confirming the porous nature of the material and indicating the presence of mesoporous structures. The composite achieves a Brunauer–Emmett–Teller (BET) specific surface area of 101.89 m^2^ g^−1^. Analysis of the BJH plot further reveals that the majority of pores fall within the mesoporous region (Fig. 5b), while the cumulative pore volume also provides evidence of micropores. The synergistic presence of micropores and mesopores is particularly beneficial, as micropores increase the available surface area for charge storage, while mesopores promote rapid electrolyte ion diffusion, together resulting in superior electrochemical performance of the supercapacitor.

**Fig. 6:**
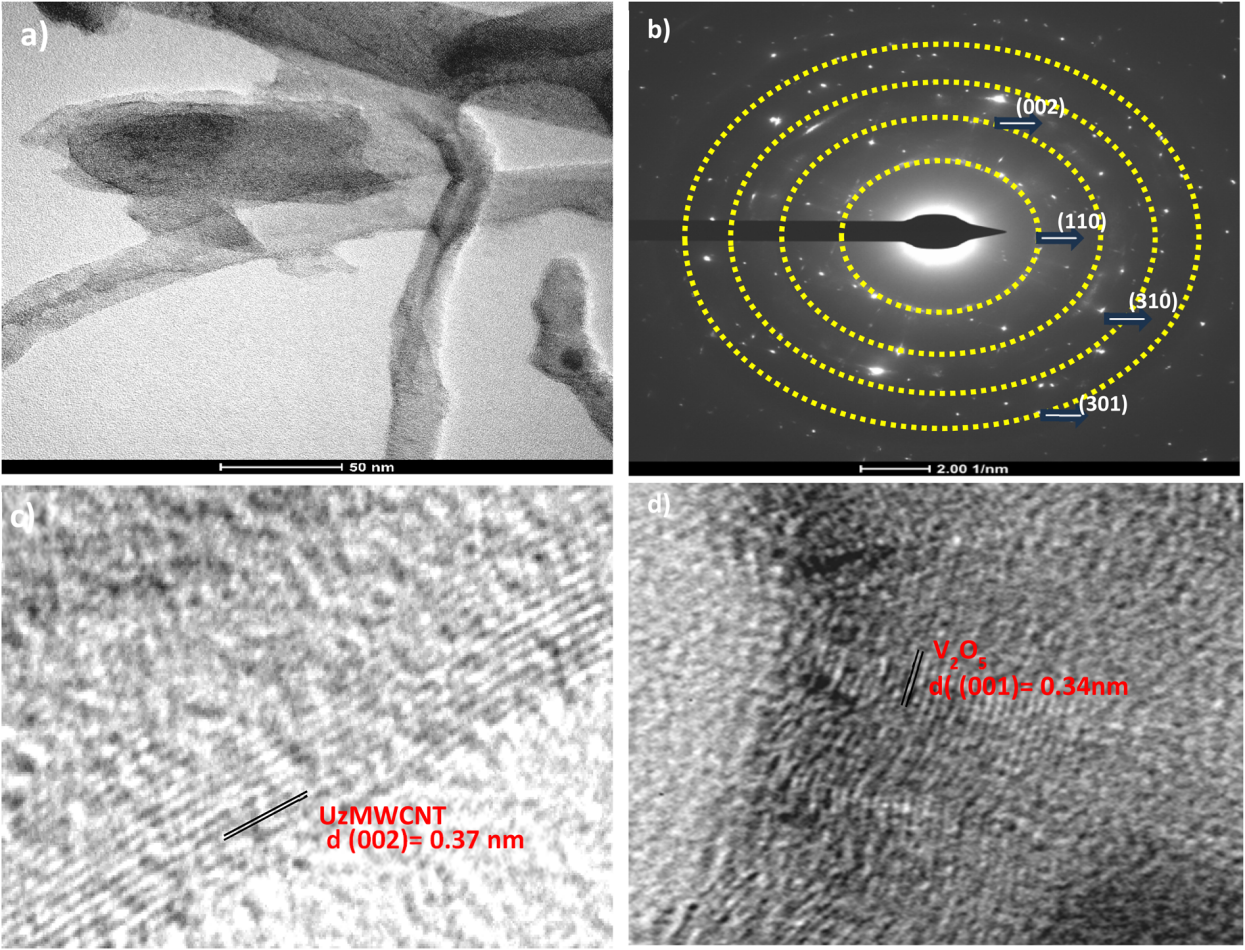
HRTEM micrographs of (a) UzMWCNT/V_2_O_5_ composite, (b) SAED pattern confirming polycrystalline characteristics of the integrated composite, (c), and (d) depict the lattice fringe of UzMWCNT/V_2_O_5_ composite.

High-resolution transmission electron microscopy (HRTEM) images of the UzMWCNT/V_2_O_5_ composite, as shown in Fig. 6a–d, provide an in-depth structural analysis and highlight its crystallographic features. The V_2_O_5_ nanoparticles are distributed across the unzipped multi-walled carbon nanotube (UzMWCNT) surface, exhibiting strong synergy that facilitates the formation of a conductive network. Additionally, the selected area electron diffraction (SAED) pattern (Fig. 6b) reveals a characteristic ring-like diffraction pattern. This indicates the polycrystalline nature of the composite. The D value of the unzipped multi-walled carbon nanotube (UzMWCNT) is 0.37 nm,^24^ as shown in Fig. 6c, which aligns with the XRD results. The interplanar spacing of 0.34 nm corresponds to the (110) plane of orthorhombic V_2_O_5_ (Fig. 6d), as confirmed by X-ray diffraction (XRD) analysis.^34^

### Electrochemical studies

**Fig. 7:**
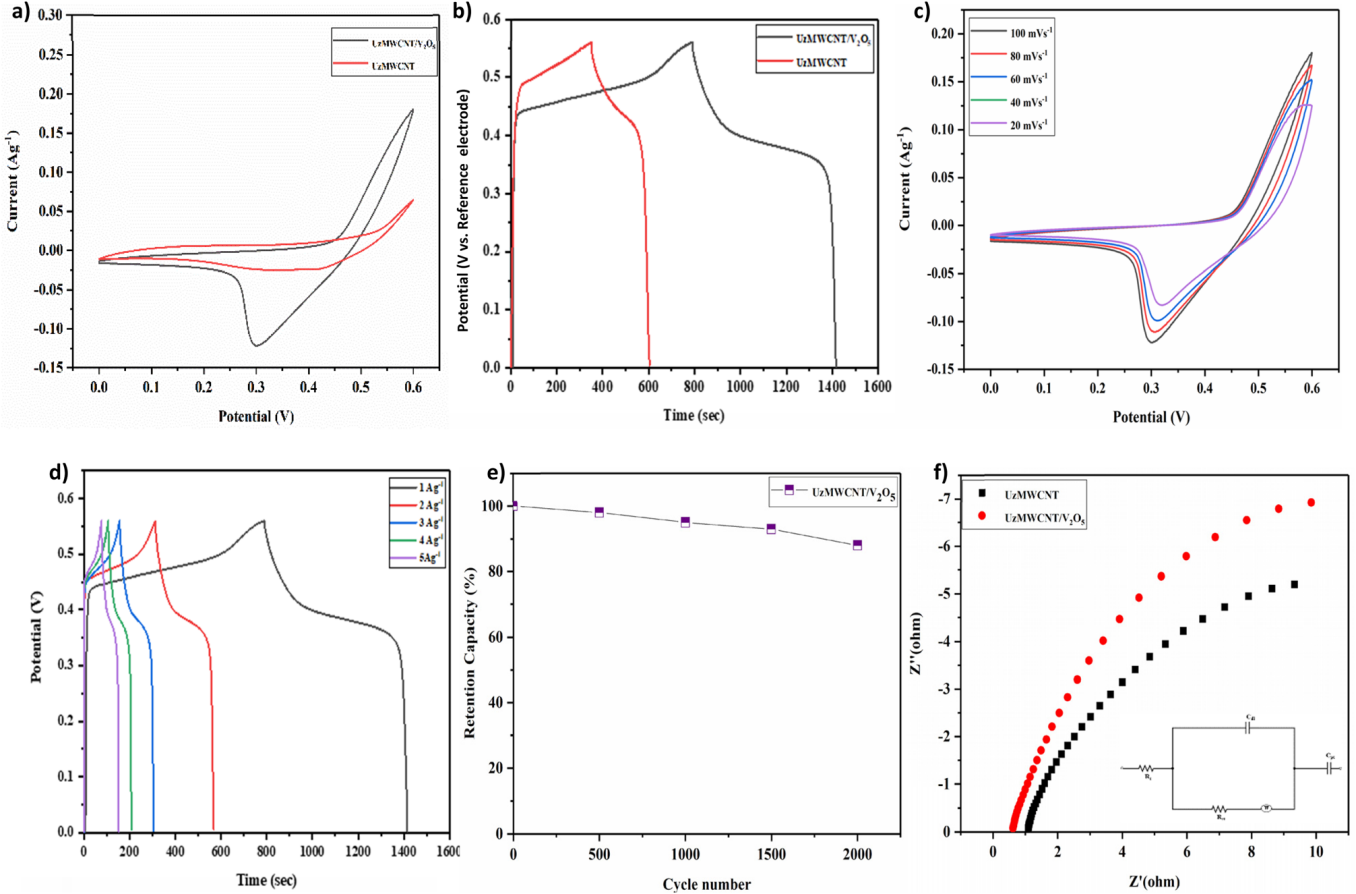
Electrochemical evaluation of UzMWCNT/V_2_O_5_ composite: (a) CV comparison of UzMWCNT/V_2_O_5_ and UzMWCNT, (b) GCD profiles of UzMWCNT/V_2_O_5_*vs.* UzMWCNT, (c) scan rate-dependent CV curves (20–100 mV s^−1^) demonstrating rate capability and reversibility, (d) GCD at varying current densities (1 to 5 A g^−1^), (e) cyclic stability analysis showing excellent capacitance and reversibility, (f) EIS comparison of UzMWCNT/V_2_O_5_ and UzMWCNT.

The electrochemical behavior of the UzMWCNT and UzMWCNT/V_2_O_5_ composite electrodes was systematically investigated in a 1 M KOH aqueous electrolyte using a three-electrode configuration. In this setup, the working electrode was fabricated using the synthesized materials, while a platinum wire and Ag/AgCl electrode served as the counter and reference electrodes, respectively. All electrochemical measurements, including CV, GCD, and EIS, were conducted within a potential window of 0 to 0.6 V to evaluate the capacitive properties of the materials. CV analysis (Fig. 7a, c, S.3. and S.4.) revealed a notable enhancement in the current response and areal capacitance for the UzMWCNT/V_2_O_5_ composite compared to the pristine UzMWCNT, V_2_O_5_ electrode. The improved electrochemical activity of the UzMWCNT/V_2_O_5_ hybrid composite is caused by the synergistic collaboration between the redox-active vanadium pentoxide and the conductive carbon structure of UzMWCNT. V_2_O_5_, a transition metal oxide with a high theoretical capacitance value, contributes significantly through its multiple reversible redox transitions (V^5+^/V^4+^), thus enhancing active sites for faradaic charge storage.

This pseudocapacitive contribution complements the electric double-layer capacitance (EDLC) behavior of pristine UzMWCNT, offering high surface area and excellent electrical conductivity. Based on the analysis, the calculated areal capacitance of the fabricated UzMWCNT/V_2_O_5_ electrode was determined to be 400 mF cm^−2^, while the electrochemical active surface area of the fabricated UzMWCNT electrode was found to be 203 mF cm^−2^. Additionally, the electrochemical performance data of MWCNTs in their zipped form are shown in (Fig. S10). These results clearly show that the unzipping process greatly improves the charge storage capacity of MWCNTs, underscoring the central purpose of this work. These results clearly demonstrate the enhanced surface activity achieved through the incorporation of V_2_O_5_ into the UzMWCNT framework.

Further insights were obtained by performing CV measurements at different scan rates (20, 40, 60, 80, and 100 mV s^−1^), as shown in Fig. 7c. Both materials demonstrated increasing current responses with higher scan rates, indicative of good capacitive behavior and efficient charge propagation. Remarkably, the UzMWCNT/V_2_O_5_ composite retained its quasi-rectangular CV shape across all scan rates, suggesting excellent electrochemical stability and rapid ion transport kinetics. Moreover, all CV curves showed clear redox peaks, confirming the pseudocapacitive character of the electrode materials. The peaks result from faradaic reactions at the interface between the electrode and electrolyte, specifically involving reversible redox interactions between hydroxyl ions (OH^−^) in the KOH electrolyte and the electroactive sites of the materials.

The electrochemical reactions can be described by the following equation:^35,36^V_2_O_5_ + *y*K^+^+ *y*e^−^ ⇌ V_2_O_5_K_*y*_Here, K^+^ denotes the metal cation involved, and *y* represents the molar ratio of metal ions present in the electrolyte solution.

At higher scan rates, redox peak intensity increased progressively with a slight positive shift in peak position. This behavior reflects the fast and reversible redox kinetics of the composite, further indicating its suitability for high-performance energy storage devices. These findings collectively point to the enhanced capacitive properties, redox activity, and stability of the UzMWCNT/V_2_O_5_ composite electrode over the pristine UzMWCNT and V_2_O_5_, positioning it as a strong candidate for advanced SC systems.^37^

The GCD measurements were carried out in 1 M KOH electrolyte over a potential window of 0 to 0.56 V to evaluate the charge storage performance of bare UzMWCNT and the binary UzMWCNT/V_2_O_5_ composite electrodes. At a CD of 1 A g^−1^, the UzMWCNT/V_2_O_5_ composite exhibited a remarkable *C*_s_ of 1135 F g^−1^, significantly surpassing that of both pristine UzMWCNT and pure V_2_O_5_ electrodes as shown in Fig. 7b., S.5. and S.6. This enhancement is attributed to the effective integration of V_2_O_5_ into the UzMWCNT matrix, which leads to a noticeable increase in discharge time, as a result of faradaic redox reaction contributions.

The Cs of the electrode materials was determined by applying eqn (1) for galvanostatic charge–discharge (GCD) analyses.^37,38^1
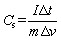
where *I* is the discharge current, Δ*t* is the discharge time, *m* is the mass of the active material, and Δ*v* is the potential window.

The improved electrochemical behavior can be ascribed to the synergistic effect between the pseudocapacitive V_2_O_5_ and the conductive carbon scaffold provided by the UzMWCNTs. This hybrid architecture enhances both ionic diffusion and electron transport pathways, resulting in efficient utilization of electroactive sites and prolonged charge–discharge cycles. The slight non-linearity in the GCD profiles of the composite electrodes further supports the presence of pseudocapacitive behavior, confirming the redox contributions of V_2_O_5_ to the overall capacitance. Fig. 7d presents the GCD curves of the UzMWCNT/V_2_O_5_ composite recorded at varying current densities ranging from 1 A g^−1^ to 5 A g^−1^. As expected, a typical trend of decreasing discharge time and *C*_s_ with increasing current density (CD) is observed, due to limited ion diffusion at higher rates, mirroring the behavior seen in CV studies with increasing scan rates.^39^ The minimal IR drop observed in UzMWCNT/V_2_O_5_ indicates excellent pseudocapacitive behavior, alongside strong electrical double-layer capacitance properties.^40^ In addition, the curves of the GCD retain quasi-triangular symmetry, indicating good capacitive reversibility and structural stability for a range of current densities.

Generally, the enhanced performance of the UzMWCNT/V_2_O_5_ electrode can be attributed not only to the high electric conductivity and porosity of the UzMWCNT network but also to the high pseudocapacitive contribution of V_2_O_5_, which offers accessible redox-active sites. This synergistic combination allows for effective charge storage by both EDLC and faradaic mechanisms, leading to high Cs and rate capability.^41^

The cycling stability of the UzMWCNT/V_2_O_5_ electrode was assessed by repeated galvanostatic charge–discharge (GCD) measurements at a constant CD of 30 mA cm^−2^ over 2000 consecutive cycles. As depicted in Fig. 7e, the electrode exhibited a gradual and consistent retention of *C*_s_, maintaining approximately 88% of its initial value after prolonged cycling. This excellent retention behavior underscores the robust structural integrity and electrochemical durability of the composite. The minimal performance degradation confirms the UzMWCNT/V_2_O_5_ material's capability to withstand continuous redox cycling, making it a promising candidate for long-term SC applications.

EIS was conducted to analyze the internal resistance and charge transport behavior of the pure UzMWCNT and the binary UzMWCNT/V_2_O_5_ composite electrodes in 1 M KOH electrolyte. The fitting process for EIS measurements employs a systematic approach based on a modified Randles circuit model, with impedance data across various frequencies, followed by complex non-linear least squares fitting to extract important characteristics. High-frequency data determines equivalent series resistance (*R*_s_), whereas mid-frequency semicircle analysis yields charge transfer resistance (*R*_ct_) and double layer capacitance (*C*_dl_). Furthermore, the Warburg-like capacitance (*C*_w_) is obtained from a low-frequency area, with fit quality evaluated using chi-square calculations. The Nyquist plots (Fig. 7f) for both samples reveal a steep slope in the low-frequency region and the absence of a pronounced semicircle in the high-frequency region.^17^ The lack of a sharp semicircle in both spectra indicates minimal charge transfer resistance (*R*_ct_) at the electrode–electrolyte interface. This indicates rapid and efficient interfacial redox kinetics, especially in the UzMWCNT/V_2_O_5_ composite, which benefits from the high conductivity of the carbon framework and the pseudocapacitive contribution from V_2_O_5_. The low-frequency region for both electrodes exhibits a near-vertical line, which is very close to the trend of an ideal capacitor. The steeper slope observed for the UzMWCNT/V_2_O_5_ composite relative to the pure UzMWCNT shows increased capacitive performance and more effective ion diffusion within the electrode matrix. Notably, the UzMWCNT/V_2_O_5_ composite also demonstrates a lower intercept on the real axis (*Z*′), suggesting a reduced equivalent series resistance (*R*_s_).^42,43^ This reduction in *R*_s_ implies improved electronic conductivity and better electrolyte accessibility due to the synergistic effect between the unzipped carbon nanotube network and the embedded V_2_O_5_ nanostructures. The EIS data reinforce that the introduction of V_2_O_5_ into the UzMWCNT framework greatly improves the electrochemical performance by lowering internal resistance and promoting near-ideal capacitive behavior, both of which are critical for high-performance SC applications.

### Electrochemical performance of fabricated symmetric supercapacitor

**Fig. 8:**
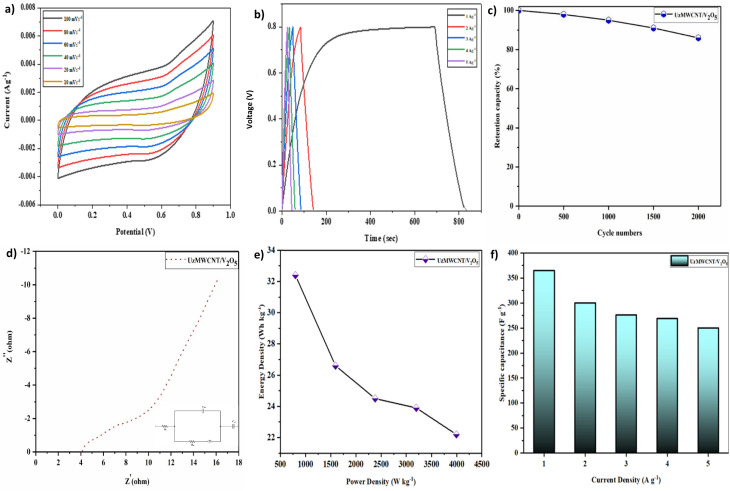
Electrochemical performance of symmetric UzMWCNT/V_2_O_5_ supercapacitor: (a) cyclic voltammetry curves at scan rates ranging from 10 to 100 mVs^−1^, (b) GCD profiles at current densities of 1 to 5 A g^−1^, (c) capacitance retention, (d) EIS of symmetric UzMWCNT/V_2_O_5_, (e) Ragone plot correlating energy density and power density with enhanced performance metrics, and (f) variation of specific capacitance with current density demonstrating excellent rate capability.

A symmetric SC was constructed using identical UzMWCNT/V_2_O_5_ composite electrodes as both the cathode and anode, as shown in Fig. 8a and b. The device was operated within an optimized potential window of 0–0.9 V to ensure maximum performance.

Moreover, the SEM-EDX obtained for the binary UzMWCNT/V_2_O_5_ composite electrodes after the electrochemical stability measurement is shown in Fig. S8, confirming the continued presence of V, C, and O elements, validating the retention of the composites' composition and stability after electrochemical cycling.

The CV profiles recorded at scan rates from 10 to 100 mV s^−1^ (Fig. 8a.) display quasi-rectangular shapes, indicative of a combination of EDLC and pseudocapacitive behavior. This hybrid characteristic results from the synergy between the conductive UzMWCNT matrix (EDLC contribution) and the redox-active V_2_O_5_ (pseudo capacitance). The progressive increase in current response with scan rate confirms efficient charge storage kinetics and good rate capability.

GCD curves obtained at current densities from 1 to 4 A g^−1^ (Fig. 8b) exhibit nearly symmetrical and linear profiles, reflecting good reversibility and capacitive nature. The *C*_s_ of the device was calculated using the formula (2):^44,45^2
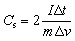
where *I* is the constant current, Δ*t* is the discharge time, *m* is the mass of a single electrode, and Δ*v* is the voltage window.

The *C*_s_ values of the binary hybrid composite of UzMWCNT/V_2_O_5_ were determined to be 365.0, 300.0, 276, 269, and 250 F g^−1^ at current densities of 1, 2, 3, 4, and 5 A g^−1^, respectively.

At 1 A g^−1^, the device achieved an ED of 33 W h kg^−1^ and a PD of 809 W kg^−1^, derived from the following expressions (3) and (4):^46,47^3
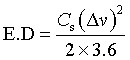
4
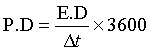


The device demonstrated excellent cyclic stability over 2000 CV cycles, retaining about 86% of its initial capacitance (Fig. 8c), highlighting its long-term operational durability, illustrating consistent performance throughout extended cycling.

EIS analysis (Fig. 8d) confirmed the low charge-transfer resistance of approximately 4.15 Ω, indicating efficient ion diffusion and superior electrical conductivity. The equivalent circuit model, provided in the inset, further supports the reliability of the impedance analysis.

The Ragone plot (Fig. 8e) summarises the relationship between PD and ED, positioning the UzMWCNT/V_2_O_5_-based hybrid for superior electrochemical energy storage systems, with a strong balance between rapid energy delivery and substantial energy storage capacity. Fig. 8f depicts the variation of specific capacitance with current density, demonstrating rate capability. Table 1 depicts the V_2_O_5_-based carbon composites for electrochemical performance.

**Table 1:** Depicts V_2_O_5_-based carbon composites for electrochemical performance

Composite	Electrolyte	Capacitance (F g^−1^)	Energy density (W h kg^−1^)	Cycling stability	References
V_2_O_5_/CNT-super activated carbon	2 M, NaNO_3_	375.5, 1 A g^−1^	—	99.5%, 200 cycles	4
V_2_O_5_ nanofibers/graphene	3 M, LiTFSI	218, 1 A g^−1^	22	—	48
PEDOP/V_2_O_5_ nanobelt	1 M, LiClO_4_	224, 1 A g^−1^	223	90%, 5000 cycles	49
V_2_O_5_/graphene/MWCNT	1 M, Na_2_SO_4_	504, 0.5 A g^−1^	70	82.9%, 32 500 cycles	50
V_2_O_5_/polypyrrole/graphene oxide	0.5 M Na_2_SO_4_	750, 5 A g^−1^	27.6	83%, 3000 cycles	51
V_2_O_5_/rGO	1 M, Na_2_SO_4_	780,1 A g^−1^	—	95%, 5000 cycles	37
Graphene nanoribbon/V_2_O_5_ nanostrip	0.5 M, Na_2_SO_4_	388, 1 A g^−1^	13.4	95.2%, 10 000 cycles	52
BiVO_4_/V_2_O_5_	1 M, KOH	701,1 A g^−1^	—	95.4%, 5000 cycles	38
g-C_3_N_4_/V_2_O_5_/PANI	1 M, KOH	880, 1 A g^−1^	16.7	78%, 2000 cycles	45
UzMWCNT/V_2_O_5_	1 M, KOH	1135, 1 A g^−1^	33	88%, 2000 cycles	This work

## Conclusions

This study successfully demonstrates that the unzipping of multiwalled carbon nanotubes (UzMWCNTs) resulted in the formation of a partially curved graphene-like structure. The incorporation of vanadium pentoxide (V_2_O_5_) nanoparticles onto this unzipped multiwalled carbon nanotube (UzMWCNT) has resulted in a composite with superior electrochemical performance. The synergistic effects arise from two key mechanisms: the enlarged diameter and partially open structure of UzMWCNTs facilitate improved electrolyte penetration and ion transport kinetics, thereby improving the charge storage capacity and rate performance. Secondly, V_2_O_5_ nanoparticles act as effective spacers, preventing agglomeration of UzMWCNT and preserving a high surface area for efficient active material utilization. This composite architecture successfully addresses common challenges in carbon-based electrodes, including graphene-like structure restacking and limited electrolyte accessibility in densely packed configurations. Future research must prioritize improving cycling stability, the primary barrier to the practical implementation of this composite system. Key approaches should include structural optimization through controlled functionalization of UzMWCNT surfaces for stronger interfacial bonding with V_2_O_5_ nanoparticles. Additionally, the choice of electrolyte with advanced formulations for better composite interface compatibility, surface chemistry modifications to enhance component adhesion and reduce active material dissolution, and heteroatom doping strategies to improve both conductivity and structural stability will be crucial. Developing scalable synthesis methods while maintaining optimized structural characteristics and investigating binder-free electrode fabrication techniques will further advance the commercial viability of this promising energy storage material.

## Author contributions

Aswathi V. P.: conceptualization, methodology, writing-original draft, Vidya Raman: conceptualization, writing – review and editing, Sreeja P. B.: methodology, supervision, validation.

## Conflicts of interest

The authors declare no conflict of interest.

## Supplementary Material

RA-016-D6RA00149A-s001

## Data Availability

Data will be made available on request. Supplementary information (SI) is available. See DOI: https://doi.org/10.1039/d6ra00149a.
