# Supplementary material for: Defect engineered unzipped multiwalled carbon nanotube/vanadium pentoxide composite for high-performance supercapacitor application
Source: RSC Adv. 2026 Feb 27;16(13):11404–14. doi: 10.1039/d6ra00149a (PMC12947306; doi:10.1039/d6ra00149a)
Supplement: RA-016-D6RA00149A-s001 [file RA-016-D6RA00149A-s001.pdf]

Supplementary Information (SI) for RSC Advances.
This journal is © The Royal Society of Chemistry 2026

Aswathi V P[a], Vidya Raman[b], Sreeja P B[a]*

[a]*Centre for Renewable Energy and Environmental Sustainability, Department of Chemistry, CHRIST University Bengaluru, Karnataka 560029, India,*

[b] *Department of Chemistry, T.M Jacob Memorial Government College, Manimalakkunu, Koothattukulam, Kerala, 686662, India*

*Sreeja.pb@christuniversity.in

https://orcid.org/0000-0002-2106-7867

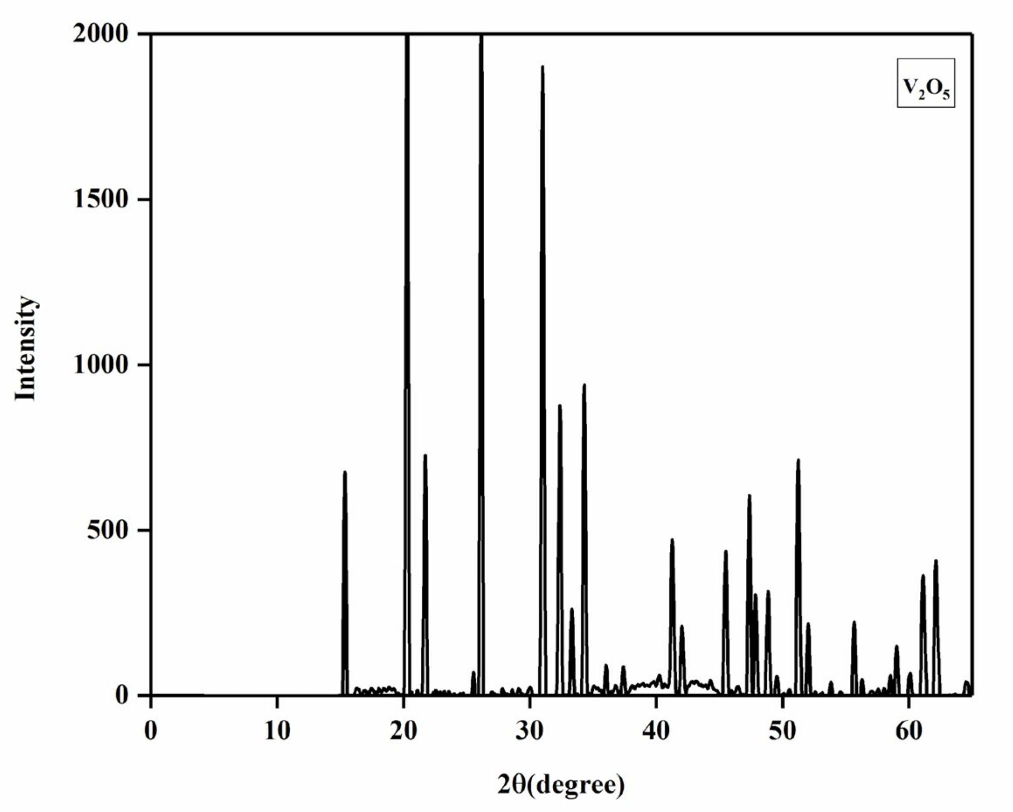

Fig. S.1. XRD spectrum of V_2O_5

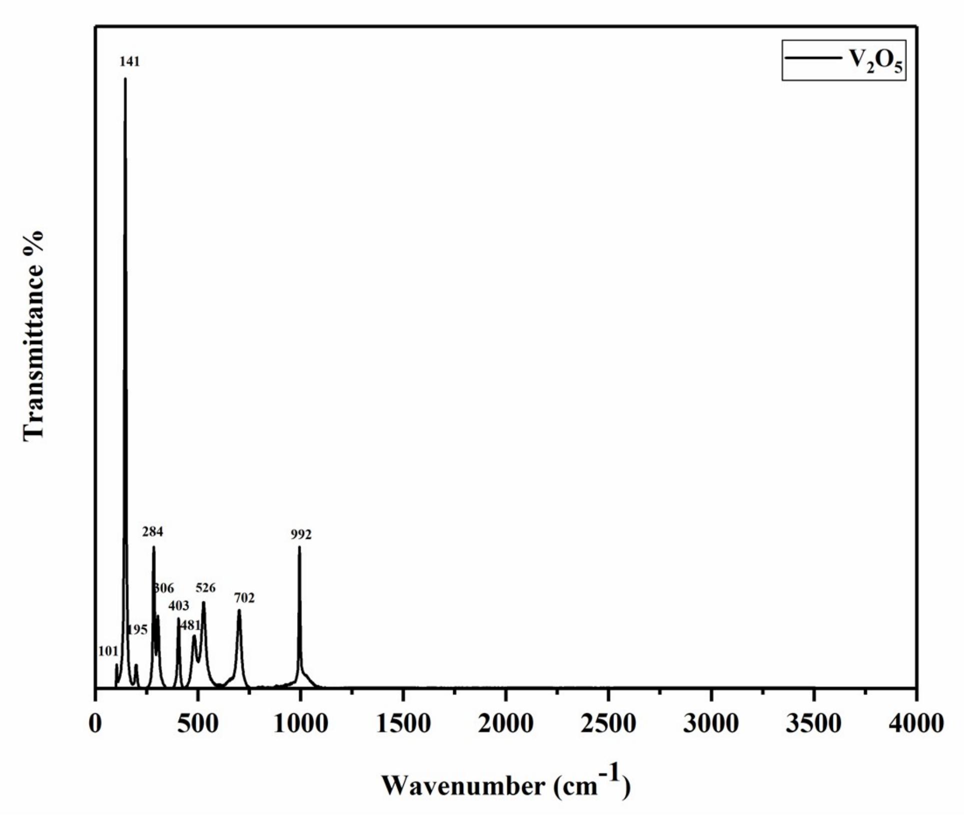

Fig.S.2. Raman Spectra of V_2O_5

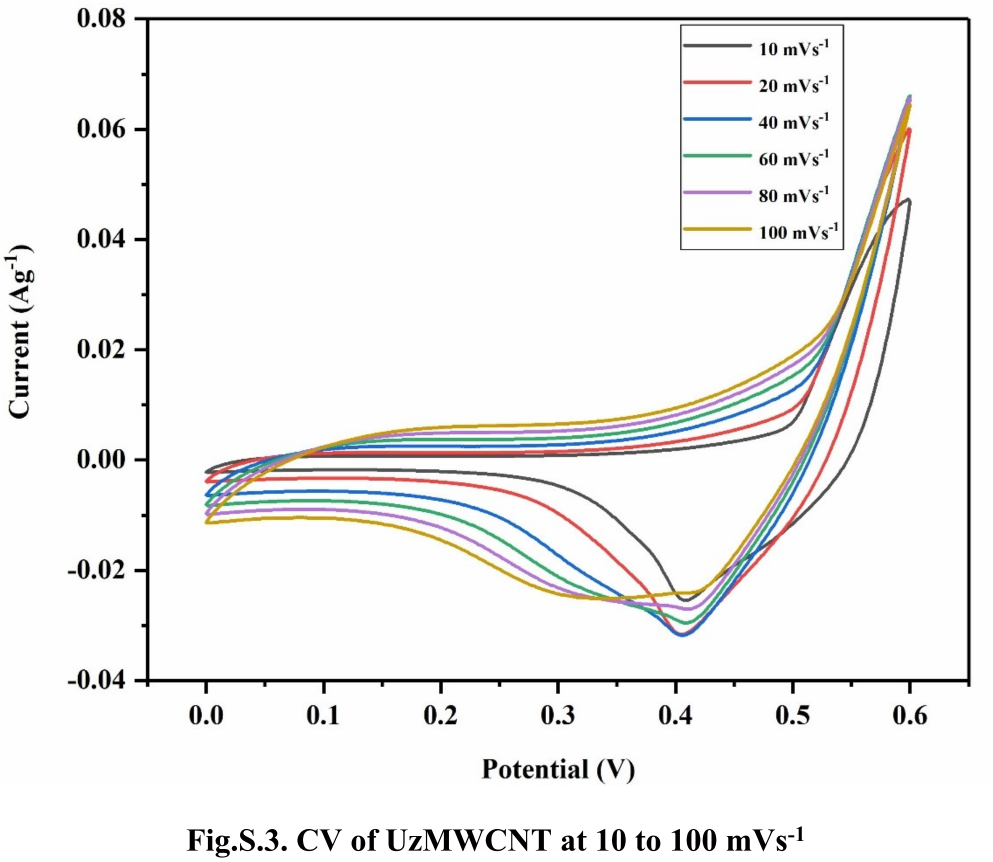

Fig.S.3. CV of UzMWCNT at 10 to 100 mVs^{-1}

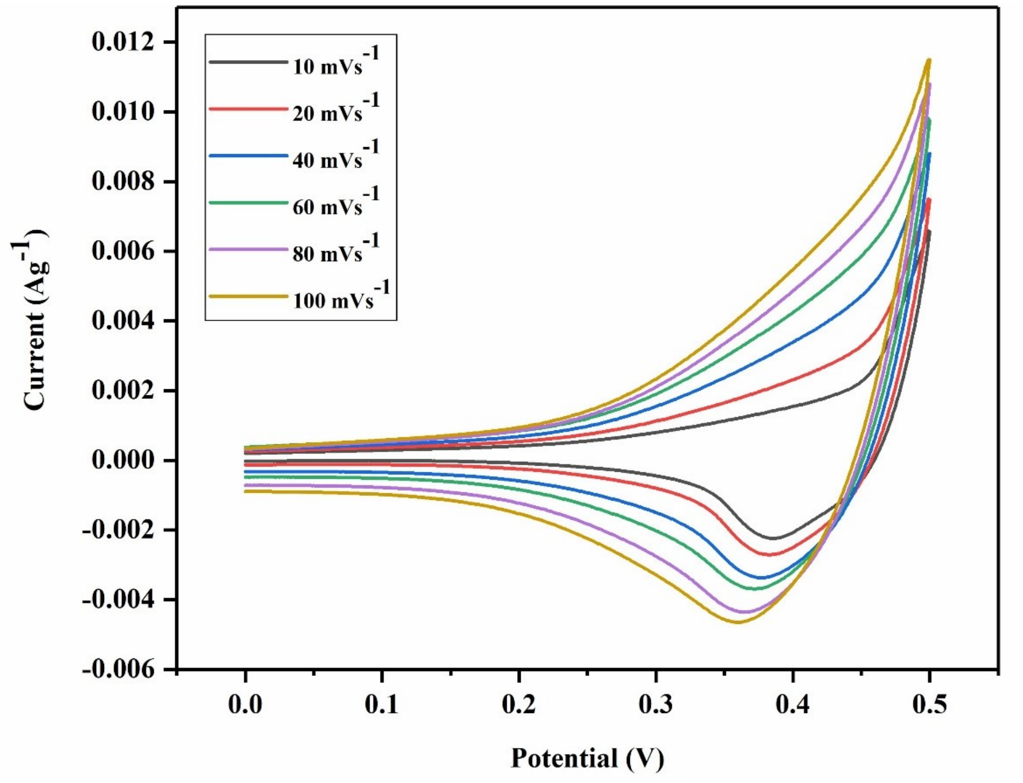

Fig.S.4. CV of V_2O_5 at 10 to 100 mVs^{-1}

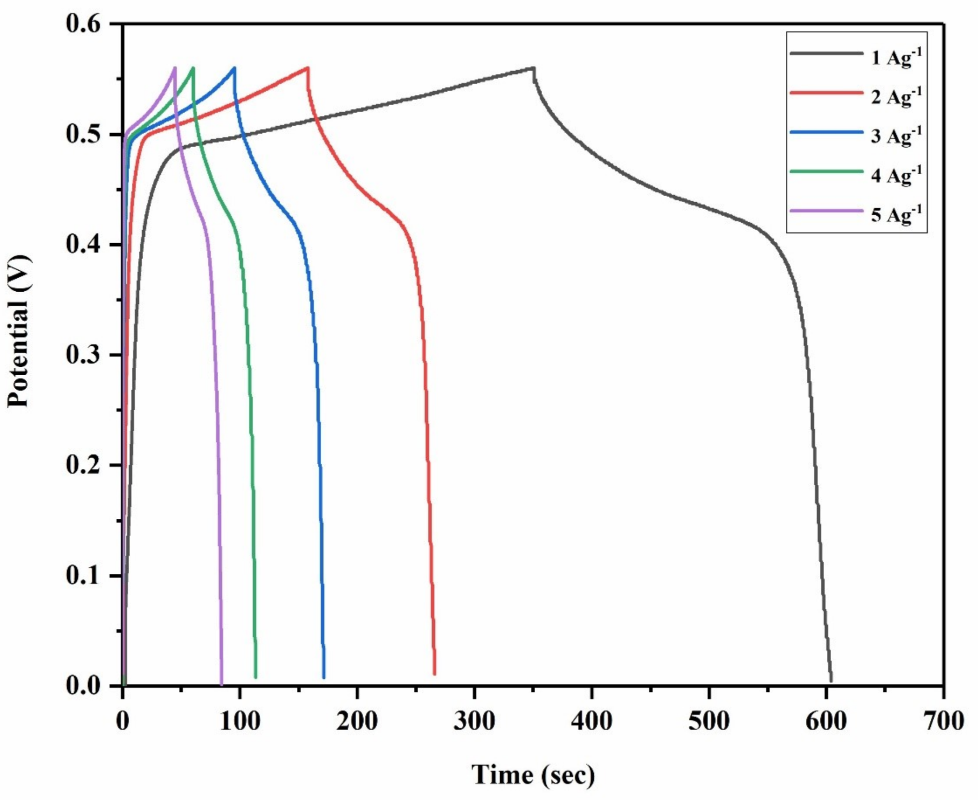

Fig.S.5. GCD of UzMWCNT at current density of 1 to 5 A g^{-1}

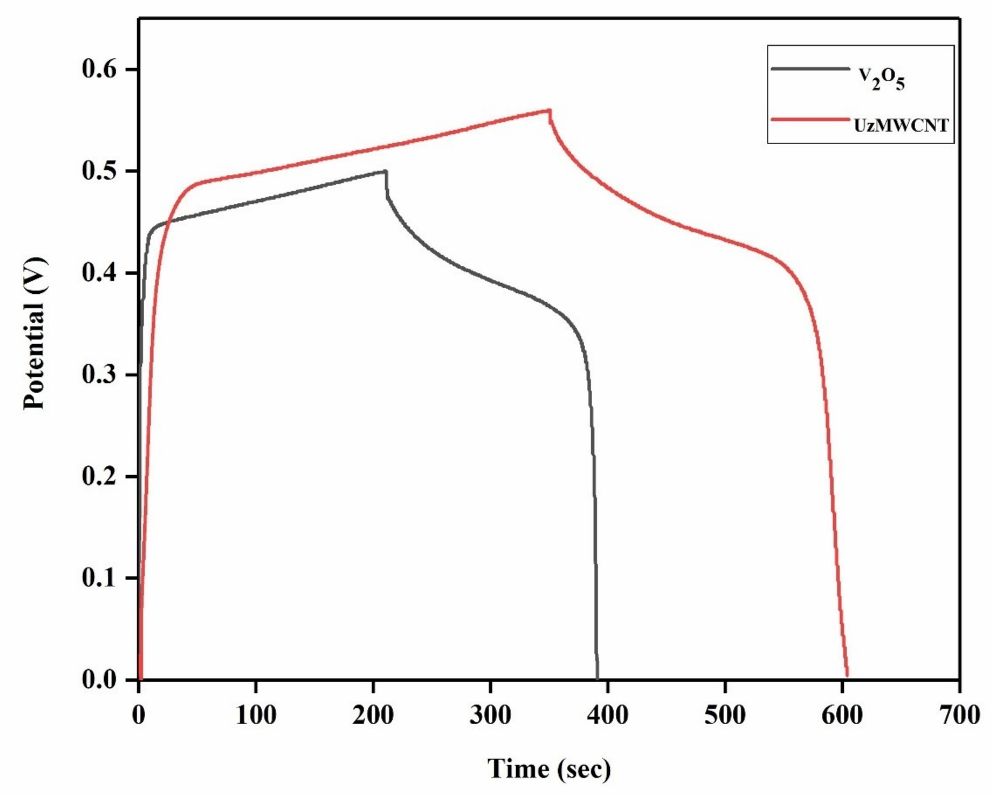

Fig.S.6. GCD of V_2O_5 and UzMWCNT

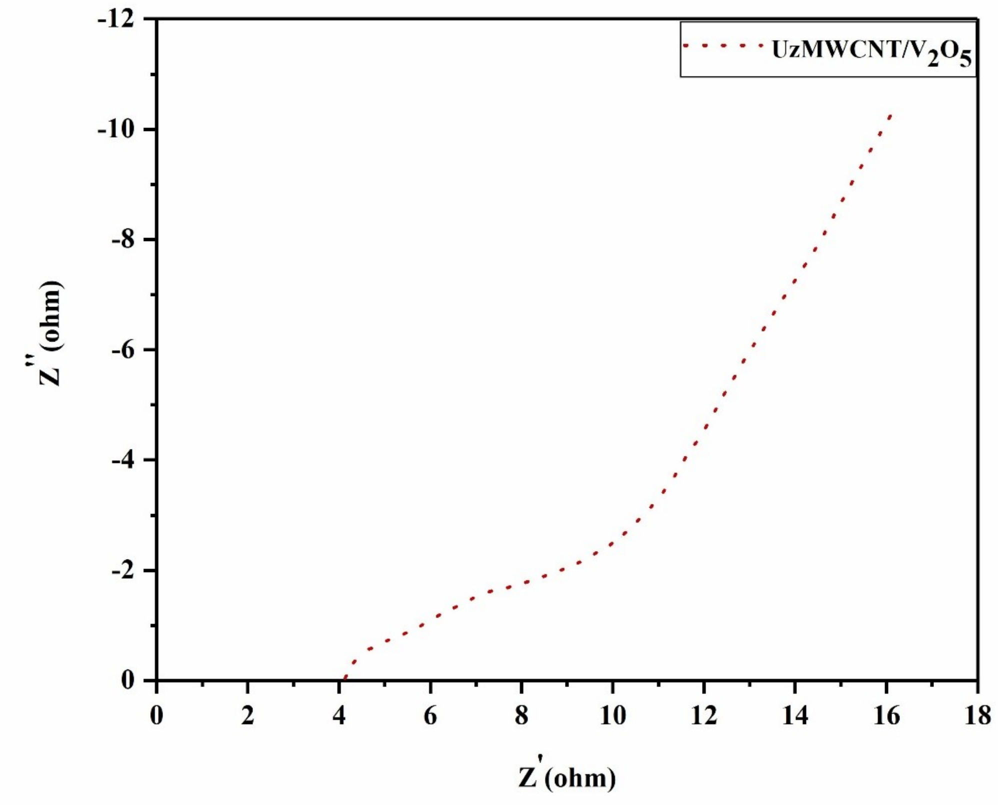

Fig.S.7. EIS of Symmetric UzMWCNT/V_2O_5

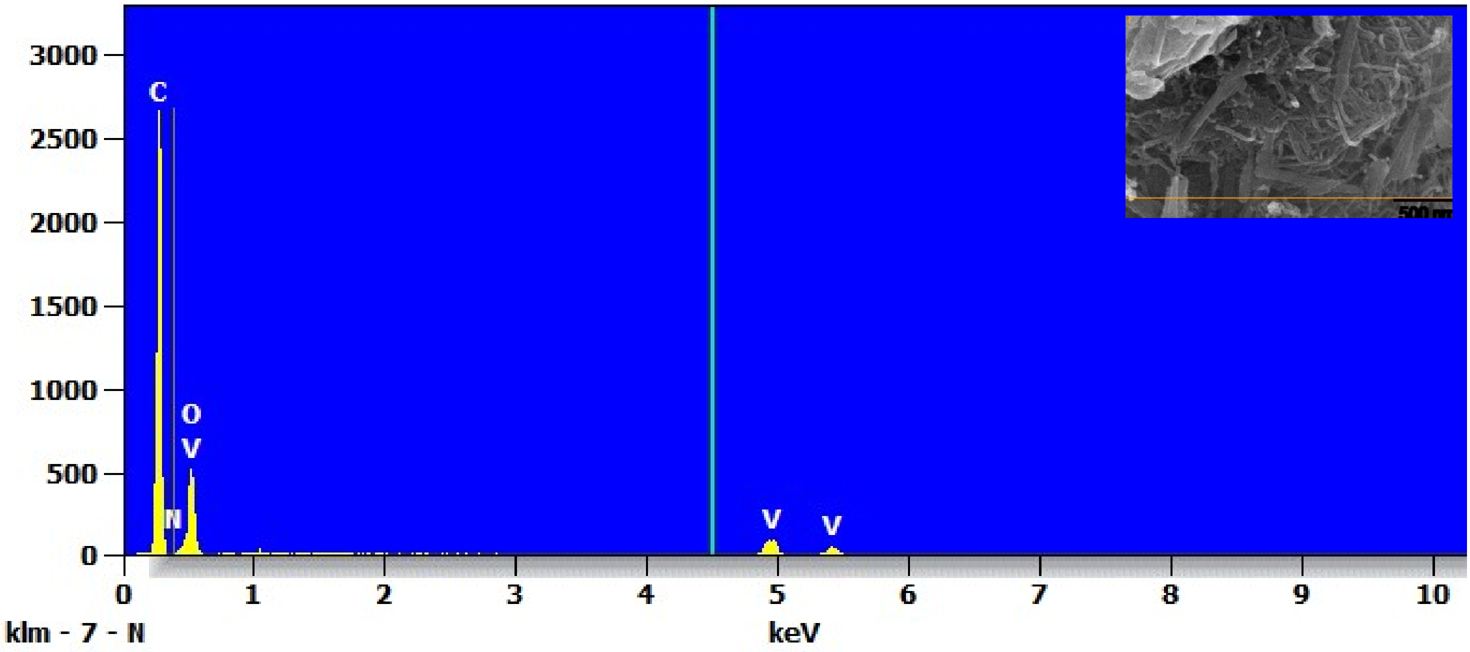

Fig.S.8 SEM-EDX after electrochemical stability measurement

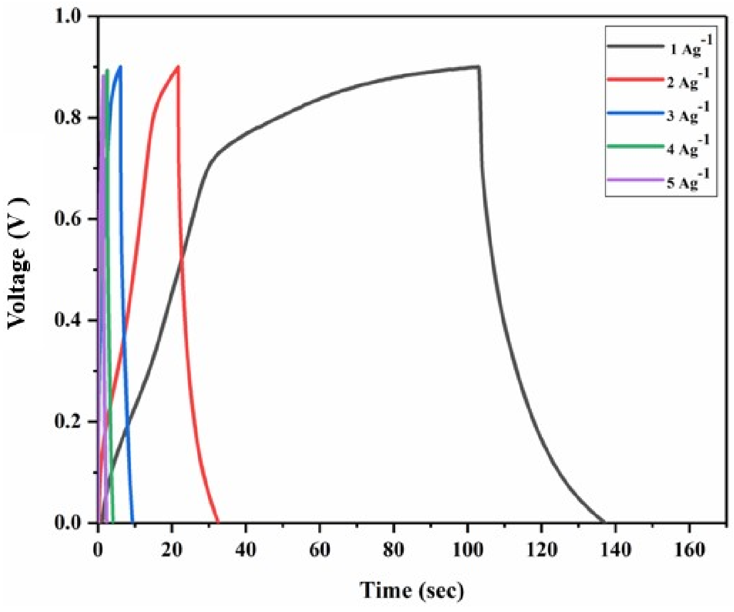

Fig.S.9. GCD of Symmetric UzMWCNT/V_2O_5 of potential window (0-0.9V)

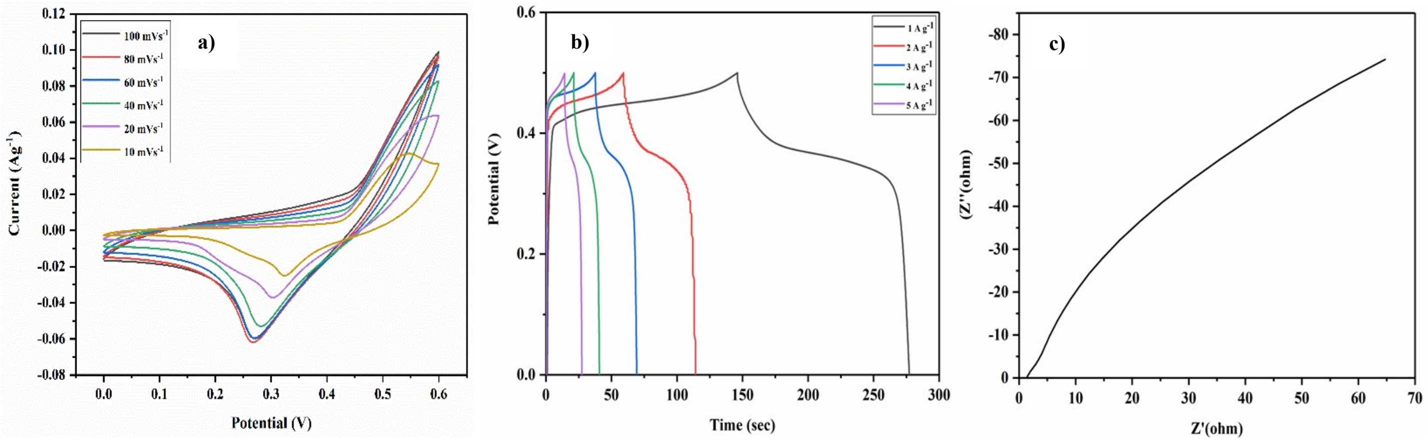

Fig.S.10 Electrochemical performance of MWCNT a) CV, b) GCD, and c) EIS
